# Oral contraceptive use and reproductive factors and risk of ovarian cancer in the European Prospective Investigation into Cancer and Nutrition

**DOI:** 10.1038/bjc.2011.371

**Published:** 2011-09-13

**Authors:** K K Tsilidis, N E Allen, T J Key, L Dossus, A Lukanova, K Bakken, E Lund, A Fournier, K Overvad, L Hansen, A Tjønneland, V Fedirko, S Rinaldi, I Romieu, F Clavel-Chapelon, P Engel, R Kaaks, M Schütze, A Steffen, C Bamia, A Trichopoulou, D Zylis, G Masala, V Pala, R Galasso, R Tumino, C Sacerdote, H B Bueno-de-Mesquita, F J B van Duijnhoven, M G M Braem, N C Onland-Moret, I T Gram, L Rodríguez, N Travier, M-J Sánchez, J M Huerta, E Ardanaz, N Larrañaga, K Jirström, J Manjer, A Idahl, N Ohlson, K-T Khaw, N Wareham, T Mouw, T Norat, E Riboli

**Affiliations:** 1 Cancer Epidemiology Unit, Nuffield Department of Clinical Medicine, University of Oxford, Oxford, UK; 2 Division of Cancer Epidemiology, German Cancer Research Center, Heidelberg, Germany; 3 Department of Community Medicine, University of Tromsø, Tromsø, Norway; 4 Inserm, Centre for Research in Epidemiology and Population Health, U1018, Institut Gustave Roussy, F-94805, Villejuif, France; 5 Paris South University, UMRS 1018, F-94805, Villejuif, France; 6 Department of Epidemiology, School of Public Health, Aarhus University, Aarhus, Denmark; 7 Institute of Cancer Epidemiology, Danish Cancer Society, Copenhagen, Denmark; 8 International Agency for Research on Cancer, Lyon, France; 9 Department of Epidemiology, German Institute of Human Nutrition, Potsdam, Germany; 10 WHO Collaborating Center for Food and Nutrition Policies, Department of Hygiene, Epidemiology and Medical Statistics, University of Athens Medical School, Athens, Greece; 11 Hellenic Health Foundation, Athens, Greece; 12 Molecular and Nutritional Epidemiology Unit, Cancer Research and Prevention Institute (ISPO), Florence, Italy; 13 Nutritional Epidemiology Unit, National Cancer Institute, Milan, Italy; 14 Istituto di Ricovero e Cura a Carattere Scientifico – Centro di Riferimento Oncologico di Basilicata, Unit of Clinical Epidemiology, Biostatistics and Cancer Registry, Rionero in Vulture, Italy; 15 Cancer Registry and Histopathology Unit, ‘Civile M.P.Arezzo’ Hospital, Ragusa, Italy; 16 Center for Cancer Prevention (CPO-Piemonte) and Human Genetic Foundation, Torino, Italy; 17 National Institute for Public Health and the Environment (RIVM), Bilthoven, The Netherlands; 18 Department of Gastroenterology and Hepatology, University Medical Centre Utrecht, Utrecht, The Netherlands; 19 Julius Center for Health Sciences and Primary Care, University Medical Center Utrecht, Utrecht, The Netherlands; 20 Public Health and Participation Directorate, Health and Health Care Services Council, Asturias, Spain; 21 Unit of Nutrition, Environment and Cancer, Catalan Institute of Oncology, Barcelona, Spain; 22 Andalusian School of Public Health, Granada, Spain; 23 CIBER Epidemiología y Salud Pública (CIBERESP), Spain; 24 Department of Epidemiology, Murcia Regional Health Authority, Murcia, Spain; 25 Navarra Public Health Institute, Pamplona, Spain; 26 Public Health Department of Gipuzkoa, Basque Government, San Sebastian, Spain; 27 Center for Molecular Pathology, Skåne University Hospital, Lund University, Malmö, Sweden; 28 Department of Surgery, Skåne University Hospital, Lund University, Malmö, Sweden; 29 Department of Clinical Sciences, Obstetrics and Gynecology, Umeå University, Umeå, Sweden; 30 Department of Public Health and Clinical Medicine, Umeå University, Umeå, Sweden; 31 Department of Medical Biosciences, Pathology, Umeå University, Umeå, Sweden; 32 Clinical Gerontology Unit, University of Cambridge, Cambridge, UK; 33 Medical Research Council Epidemiology Unit, University of Cambridge, Cambridge, UK; 34 Department of Epidemiology and Biostatistics, School of Public Health, Imperial College, London, UK

**Keywords:** reproductive history, oral contraceptive use, ovarian cancer, cohort study

## Abstract

**Background:**

It is well established that parity and use of oral contraceptives reduce the risk of ovarian cancer, but the associations with other reproductive variables are less clear.

**Methods:**

We examined the associations of oral contraceptive use and reproductive factors with ovarian cancer risk in the European Prospective Investigation into Cancer and Nutrition. Among 327 396 eligible women, 878 developed ovarian cancer over an average of 9 years. Hazard ratios (HRs) and 95% confidence intervals (CIs) were estimated using Cox proportional hazard models stratified by centre and age, and adjusted for smoking status, body mass index, unilateral ovariectomy, simple hysterectomy, menopausal hormone therapy, and mutually adjusted for age at menarche, age at menopause, number of full-term pregnancies and duration of oral contraceptive use.

**Results:**

Women who used oral contraceptives for 10 or more years had a significant 45% (HR, 0.55; 95% CI, 0.41–0.75) lower risk compared with users of 1 year or less (*P*-trend, <0.01). Compared with nulliparous women, parous women had a 29% (HR, 0.71; 95% CI, 0.59–0.87) lower risk, with an 8% reduction in risk for each additional pregnancy. A high age at menopause was associated with a higher risk of ovarian cancer (>52 *vs* ⩽45 years: HR, 1.46; 95% CI, 1.06–1.99; *P*-trend, 0.02). Age at menarche, age at first full-term pregnancy, incomplete pregnancies and breastfeeding were not associated with risk.

**Conclusion:**

This study shows a strong protective association of oral contraceptives and parity with ovarian cancer risk, a higher risk with a late age at menopause, and no association with other reproductive factors.

In developed countries, ovarian cancer is the sixth most common malignancy and cause of cancer death in women (American Cancer Society, 2007). Some reproductive factors are associated with the risk of ovarian cancer, with evidence for a protective association of high parity and oral contraceptive use (Negri *et al*, 1991; Whittemore *et al*, 1992; Hankinson *et al*, 1995; Beral *et al*, 2008). However, the evidence that other reproductive variables, such as breastfeeding, incomplete pregnancies, age at menarche and age at menopause, are associated with risk is weak and inconsistent, and mostly comes from case–control studies (Riman *et al*, 2004).

The aim of this study was to evaluate the association between oral contraceptive use and reproductive factors with ovarian cancer risk in a large prospective European study. A secondary aim was to examine whether these associations differed by participant characteristics such as age at enrolment, body mass index (BMI) and other factors.

## Materials and Methods

### Source and study population

The European Prospective Investigation into Cancer and Nutrition (EPIC) includes approximately 370 000 women and 150 000 men and was designed to investigate dietary and lifestyle determinants of cancer. The participants were recruited between 1992 and 2000 in 23 centres in 10 European countries (Denmark, France, Germany, Greece, Italy, The Netherlands, Norway, Spain, Sweden and the United Kingdom). The cohort population and data collection procedures have been described in detail elsewhere (Riboli *et al*, 2002). Approval for the study was obtained from the local ethics committees in the participating countries and the internal review board of the International Agency for Research on Cancer.

Incident cancer cases were identified through linkage to population cancer registries in Denmark, Italy, The Netherlands, Norway, Spain, Sweden and the UK, or using a combination of methods including linkage to health insurance records, cancer and pathology registries, and active follow-up of study participants or their next of kin in France, Germany and Greece.

Of the approximately 370 000 women enrolled in the cohort, women were excluded if they had prevalent cancer (*n*=19 707), if they had incomplete follow-up data (*n*=2205), if they had a bilateral ovariectomy (*n*=10 500), if they did not return the baseline questionnaire (*n*=509), if they never menstruated (*n*=61), if they had missing information on oral contraceptive use and all reproductive history variables (*n*=7589), or if they were diagnosed with a non-epithelial ovarian cancer (*n*=26). Of the final analytic cohort of 327 396 women, 878 developed epithelial ovarian cancer from the date of recruitment until the end of follow-up (31 December 2003 to 20 December 2006, according to centre). The cancer diagnosis was confirmed by histology for 77.6% of the cases, by clinical examination for 13.8% and the remaining 8.6% by self-report, autopsy or death certificate. In all, 47% of tumours were of a serous histology, 10% were mucinous, 10% endometrioid, 4% clear cell and 1% undifferentiated; the remaining 28% had missing, undefined or not otherwise specified histologies. Of the 878 cases, 70 were of low malignant potential (borderline tumours); exclusion of these cases did not change the risk estimates, and they were therefore included in the final analysis.

### Exposure and covariate assessment

Women were asked at the baseline questionnaire whether they had ever used oral contraceptives, their duration of use up until the time of recruitment, and the age at first use. Information on age at menarche and menopause, numbers of full-term pregnancies (defined as the sum of live and stillbirths), incomplete pregnancies (defined as induced or spontaneous abortions) and age at the first full-term pregnancy was also collected. Information on breastfeeding was collected for the first three full-term pregnancies and the last one. The duration of breastfeeding was calculated as the sum for all pregnancies for which we had information. For women with more than four pregnancies and who had breastfed for all the pregnancies for which we had information, the duration of breastfeeding was taken to be the number of pregnancies multiplied by the mean of breastfeeding duration of each child. Menopausal status was defined according to information on menstruation status, hysterectomy, ovariectomy, use of exogenous hormones and age, details of which are provided elsewhere (Dossus *et al*, 2010). The cumulative duration of menstrual cycles among postmenopausal women was defined as the time between age at menarche and age at menopause minus the cumulative duration of full-term pregnancies (calculated as the number of full-term pregnancies times 0.75) and oral contraceptive use.

Information on anthropometric data, physical activity, smoking status, education level, self-reported diabetes mellitus, simple hysterectomy (with ovarian conservation) and unilateral ovariectomy status was also collected from the baseline questionnaire. Weight and height were measured at recruitment, except for part of the Oxford cohort, the Norwegian cohort, and approximately two-thirds of the French cohort, among whom weight and height were self-reported. Body mass index was calculated as weight in kilograms divided by height in metres squared. A combined physical activity index was calculated, which incorporated occupational and recreational activities.

### Statistical analysis

Hazard ratios (HRs) and their 95% confidence intervals (CIs) for ovarian cancer were estimated using Cox proportional hazards models with age as the underlying time scale. Entry and exit time was defined as the woman's age at recruitment and age at ovarian cancer diagnosis or censoring (death, lost to follow-up or end of follow-up), respectively. All models were stratified by EPIC recruitment centre and age at enrolment (⩽50, 51–53, 54–56, 57–59, 60–62, 63–65, >65 years). The proportionality of hazards was verified based on the slope of the Schoenfeld residuals over time, which is equivalent to testing that the log HR function is constant over time (Schoenfeld, 1982). Linear trends were tested by entering appropriate ordinal variables into the model, the coefficients of which were evaluated by the Wald test.

All models were adjusted for potential risk factors for ovarian cancer, including tobacco smoking status (never, former, current), BMI (continuous), unilateral ovariectomy (no, yes), simple hysterectomy (no, yes), menopausal hormone therapy (never, former, current use: oestrogen only, oestrogen plus progestin, other formulation), and further mutually adjusted for age at menarche (<12, 12, 13, 14, ⩾15 years), age at menopause (premenopausal, perimenopausal, postmenopausal: ⩽45, 46–50, 51–52, >52 years; based on fourths of the distribution in the controls), number of full-term pregnancies (0, 1, 2, 3, ⩾4) and duration of oral contraceptive use (never, ever: ⩽1, 2–4, 5–9, ⩾10 years), as applicable. Only the fully adjusted statistical models are presented in the text, because the centre and age-stratified models yielded almost identical estimates. For each covariate, missing values were assigned to separate categories, where appropriate; an analysis that excluded individuals with missing values for these covariates produced similar results and is not presented here. Further adjustments for physical activity, diabetes mellitus and education yielded almost identical results and are not presented.

To examine whether the oestrogen dose in oral contraceptive formulations influenced the risk, the analyses were stratified by calendar year of first oral contraceptive use. The oral contraceptives prescribed before 1970 were typically high-dose preparations, whereas those prescribed between 1970 and 1980 were typically medium dose and by 1980 most prescriptions were for low-dose preparations (Piper and Kennedy, 1987; Thorogood and Villard-Mackintosh, 1993).

Analyses were also performed by ovarian cancer histology, EPIC-participating country and according to other factors including: age at recruitment (at the median, <50 *vs* ⩾50), BMI (at the median, <24 *vs* ⩾24 kg m^−2^), smoking status (ever *vs* never), parity (parous *vs* nulliparous), menopausal status (postmenopausal *vs* pre/perimenopausal) and menopausal hormone therapy at recruitment (ever *vs* never use and current *vs* never use). Tests for interaction were carried out by using the relevant exposure variables, indicator variables for the potentially modifying factors, and product terms of the two variables. Sensitivity analyses were run after excluding ovarian cancer cases that developed in the first 2 years of follow-up or after excluding participants with a history of simple hysterectomy and/or unilateral ovariectomy at baseline. The statistical significance of the interaction terms was evaluated by the Wald test. All *P*-values (*P*) were two-sided and all analyses were performed using STATA version 11 (College Station, TX, USA).

## Results

After recruitment into the study, 878 cases of ovarian cancer were diagnosed during 2.9 million person-years of follow-up, over an average of 9 years (Table 1). The mean age at recruitment and diagnosis for the ovarian cancer cases was 55 and 60 years, respectively. In all, 59% of the cohort reported ever use of oral contraceptives (Table 1), 9% of which were current users. Ever use of oral contraceptive was lowest in Greece (10%) and highest in Germany (82%). The median duration of oral contraceptive use was 5 years but women in Greece, Italy and Spain had considerably shorter durations. Eighty percent of the cohort reported having had a full-term pregnancy, 28% reported ever having an incomplete pregnancy and 82% reported that they had ever breastfed. The overall mean ages at menarche and menopause were 13 and 50 years, respectively, without any notable variation by country.

### Oral contraceptive use

Compared with never users of oral contraceptives, ever users had a significantly lower risk of ovarian cancer in the age and centre-stratified model (HR, 0.84; 95% CI, 0.72–0.98), and that remained very similar after additional adjustment for smoking status, BMI, unilateral ovariectomy, simple hysterectomy, menopausal hormone therapy, age at menarche, age at menopause and number of full-term pregnancies (HR, 0.86; 95% CI, 0.73–1.00; Table 2). Increasing duration of oral contraceptive use was associated with a progressive reduction in risk; women who used oral contraceptives for 10 or more years had a 45% (HR, 0.55; 95% CI, 0.41–0.75) lower risk compared with users of 1 year or less, which corresponded to a 13% (HR, 0.87; 95% CI, 0.82–0.93) lower risk per 5 years of use (*P*-trend, <0.01). There was no association between age at first use of oral contraceptives and risk of ovarian cancer (Table 2), either before or after adjusting for duration of oral contraceptive use (data not shown).

The reduced risk of ovarian cancer associated with ever use of oral contraceptives was similar within each category of calendar year of first use (1960s or earlier: HR, 0.83; 95% CI, 0.68–1.01; 1970s: HR, 0.80; 95% CI, 0.63–1.01; 1980s or later: HR, 0.90; 95% CI, 0.69–1.16). The risk associated with increasing duration of oral contraceptive use did not vary significantly by ovarian cancer histology, country, age at recruitment, smoking status or parity (data not shown). However, there was some evidence that this association varied by BMI (Table 3; *P*-interaction, 0.01), with a stronger inverse association in women with a BMI <24 kg m^−2^ (⩾10 *vs* ⩽1 year of use: HR, 0.38; 95% CI, 0.25–0.59; *P*-trend, <0.01) compared with women with a BMI of 24 kg m^−2^ or more (HR, 0.80; 95% CI, 0.52–1.23; *P*-trend, 0.24). This association was also stronger in pre/perimenopausal women (Table 3; ⩾10 *vs* ⩽1 years of use: HR, 0.41; 95% CI, 0.26–0.63; *P*-trend, <0.01) than in postmenopausal women (HR, 0.76; 95% CI, 0.49–1.17; *P*-trend, 0.14; *P*-interaction, 0.02).

### Parity and other reproductive variables

Compared with nulliparous women, women who had children had a 29% (HR, 0.71; 95% CI, 0.59–0.87) lower risk of ovarian cancer (Table 4), with a progressive reduction in risk with each additional pregnancy. Compared with women with one full-term pregnancy, women with four or more full-term pregnancies had a 23% lower risk (HR, 0.77; 95% CI, 0.57–1.04), which corresponded to an 8% (HR, 0.92; 95% CI, 0.85–0.99) lower risk per full-term pregnancy (*P*-trend, 0.03). Compared with nulliparous women, women with four or more full-term pregnancies had a 38% lower risk (HR, 0.62; 95% CI, 0.46–0.83; *P*-trend, <0.01). Age at first full-term pregnancy, ever having had an incomplete pregnancy and breastfeeding were not associated with risk (Table 4).

The risk of ovarian cancer was not associated with age at menarche or menopausal status (Table 4), although age at menopause was significantly positively associated with risk (>52 *vs* ⩽45 years: HR, 1.46; 95% CI, 1.06–1.99). The estimated cumulative duration of menstrual cycles was also associated with a higher risk (>36 *vs* ⩽27 years: HR, 1.57; 95% CI, 1.16–2.13; *P*-trend, <0.01). A one-year increase in menstrual lifespan corresponded to a 2% higher risk (HR, 1.02; 95% CI, 1.01–1.04). However, when women who were diagnosed with ovarian cancer within the first 2 years of follow-up were excluded from the analysis (194 cases), the associations of age at menopause (>52 *vs* ⩽45 years: HR, 1.40; 95% CI, 0.98–2.00; *P*-trend, 0.12) and menstrual lifespan (>36 *vs* ⩽27 years: HR, 1.37; 95% CI, 0.98–1.93; *P*-trend, 0.01) with risk of ovarian cancer were attenuated.

None of the associations between reproductive factors and risk of ovarian cancer differed by ovarian cancer histology, country, age, BMI, smoking status, menopausal status or menopausal hormone therapy (data not shown). In addition, sensitivity analyses that excluded participants with a history of simple hysterectomy and/or unilateral ovariectomy at recruitment yielded almost identical estimates.

## Discussion

This large prospective study confirms the strong protective association of oral contraceptive use and parity on risk of ovarian cancer. No significant associations with risk were found for age at menarche, age at first full-term pregnancy, incomplete pregnancies or breastfeeding.

There is strong evidence that oral contraceptive use reduces ovarian cancer risk; a collaborative pooled analysis of 45 epidemiological studies (13 prospective and 32 case–control studies) reported a relative risk of 0.73 (95% CI, 0.70–0.76) for ever *vs* never users of oral contraceptives (Beral *et al*, 2008). That study also showed a progressive decline with increasing duration of use (10–14 years *vs* <1 year: HR, 0.56; 95% CI, 0.50–0.62) of a magnitude similar to our findings. The pooled analysis also showed that, although the risk is attenuated with increasing time since last use, the protective effect of oral contraceptives on ovarian cancer remains for many years after cessation of use (Beral *et al*, 2008). However, we were unable to examine this association because many EPIC centres did not collect information on the age at last oral contraceptive use, and it is possible that the stronger inverse associations observed for the duration of oral contraceptive use and ovarian cancer risk among lean and pre/perimenopausal women in our study population might, in part, reflect a shorter time since last use among these women. Our results also suggest that the dose of oestrogen contained in oral contraceptives has a negligible impact on the association with ovarian cancer risk, as shown by similar estimates in risk according to calendar year of first oral contraceptive use, and which is consistent with the findings from the pooled analysis (Beral *et al*, 2008).

A protective as sociation between parity and ovarian cancer risk has been observed in most previous studies (Negri *et al*, 1991; Whittemore *et al*, 1992; Hankinson *et al*, 1995; Vachon *et al*, 2002; Tung *et al*, 2003; Moorman *et al*, 2009). The current study suggests that there is a quite large reduction in risk with the first child, with progressive lower risks with each additional full-term pregnancy. Our finding of no association between age at first full-term pregnancy and ovarian cancer risk is consistent with most studies (Kvale *et al*, 1988; Booth *et al*, 1989; Gwinn *et al*, 1990; Risch *et al*, 1994; Hankinson *et al*, 1995; Purdie *et al*, 1995; Riman *et al*, 2002; Vachon *et al*, 2002; Braem *et al*, 2010), although some have reported a reduction in risk with an early age at first full-term pregnancy (Adami *et al*, 1994; Albrektsen *et al*, 1996; Mogren *et al*, 2001; Titus-Ernstoff *et al*, 2001; Whiteman *et al*, 2003; Pike *et al*, 2004). Our finding of no association between number of incomplete pregnancies and ovarian cancer risk is consistent with some studies (Kvale *et al*, 1988; Booth *et al*, 1989; Risch *et al*, 1994; Purdie *et al*, 1995; Titus-Ernstoff *et al*, 2001), although others have found a reduced risk with more incomplete pregnancies, but smaller in magnitude than the reduction observed for full-term pregnancies (Negri *et al*, 1991; Whittemore *et al*, 1992; Riman *et al*, 2002; Pike *et al*, 2004; Zhang *et al*, 2004).

Breastfeeding was not significantly associated with ovarian cancer risk in this study population, and although this is consistent with several studies (Booth *et al*, 1989; Purdie *et al*, 1995; Titus-Ernstoff *et al*, 2001; Riman *et al*, 2002; Pike *et al*, 2004), the cumulative duration of breastfeeding was relatively short in our study (upper category: >13 months), and we cannot rule out the possibility of a protective association with a longer duration (e.g., >18 or >24 months) as seen in some other studies (Gwinn *et al*, 1990; Whittemore *et al*, 1992; Danforth *et al*, 2007; Jordan *et al*, 2010). The association between age at menarche and menopause and the risk of ovarian cancer has been extensively studied, although the findings have generally been weak and not statistically significant (Kvale *et al*, 1988; Gwinn *et al*, 1990; Whittemore *et al*, 1992; Hankinson *et al*, 1995; Purdie *et al*, 1995; Schildkraut *et al*, 2001; Titus-Ernstoff *et al*, 2001; Riman *et al*, 2002; Pike *et al*, 2004; Jordan *et al*, 2005; Braem *et al*, 2010). The current study shows that although there is no association between age at menarche and risk, a late age at menopause and the cumulative duration of menstrual cycles are both positively associated with ovarian cancer risk. If the number of menstrual cycles is important in the development of ovarian cancer, as has been suggested (Pike *et al*, 2004), it is likely that a difference of 1 or 2 years in the age at menarche will not contribute sufficiently to the lifetime number of menstrual cycles compared with larger differences in age at menopause, parity and oral contraceptive use. However, the increased risk in ovarian cancer observed with age at menopause was restricted to women in the highest category (52 years or older), and it is possible that this increased risk is partially a result of reverse causation, whereby older women may mistake bleeding from a sub-clinical cancer as menses. Indeed, after excluding the first 2 years of follow-up, the risk associated with a relatively late age at menopause was slightly attenuated and no longer statistically significant.

The exact mechanisms through which oral contraceptives and parity may reduce risk are not known, although several hypotheses have been proposed. These include inhibition of ovulation (Fathalla , 1971), exposure to low concentrations of gonadotropins (Stadel, 1975; Cramer and Welch, 1983), reduced retrograde transportation of contaminants from the vagina or growth factors from the uterus (Harlow *et al*, 1992; Cramer and Xu, 1995) and exposure to high progesterone concentrations (Risch, 1998; Lau *et al*, 1999; Syed *et al*, 2001; Riman *et al*, 2004; Mukherjee *et al*, 2005). In particular, pregnancy leads to anovulation, reduces gonadotropin secretion, increases progesterone levels and temporarily interrupts the retrograde transportation. Similarly, combined oral contraceptives suppress the midcycle gonadotropin surge with a consequent inhibition of ovulation. The increase in risk with a late age at menopause supports the incessant ovulation and the retrograde transportation hypotheses, but is not readily compatible with the gonadotropin stimulation and progesterone deficiency hypotheses (Riman *et al*, 2002).

Strengths of the present study include its prospective design and the wide distribution of oral contraceptive use and reproductive variables in different European studies. However, although EPIC is one of the largest studies to date to examine reproductive factors with ovarian cancer risk, there was limited statistical power to detect differences by histological subtypes of ovarian cancer. All analyses were adjusted for potential risk factors of ovarian cancer and further mutually adjusted for menstrual and reproductive variables, and which made little difference to the risk estimates suggesting that residual confounding is unlikely to influence our results. In particular, we lacked information on tubal ligation in EPIC but given the low prevalence of this procedure in Europe (Riman *et al*, 2002), the extent of potential confounding is expected to be minimal.

In conclusion, this study shows a strong protective association of oral contraceptive use and parity with risk of ovarian cancer, a higher risk with a late age at menopause, and no association with other reproductive factors in this large cohort of European women.

## Figures and Tables

**Table 1:** Participant characteristics at recruitment among women in the EPIC cohort by country, means or medians (s.d. or inter-quartile ranges) and percentages

**Characteristic**	**All (*n*=327 396)**	**Denmark (*n*=27 958)**	**France (*n*=67 504)**	**Germany (*n*=27 586)**	**Greece (*n*=14 459)**	**Italy (*n*=29 857)**	**Netherlands (*n*=26 259)**	**Norway (*n*=35 800)**	**Spain (*n*=23 975)**	**Sweden (*n*=20 418)**	**UK (*n*=53 580)**
Number of cases	878	92	192	56	32	73	64	76	52	87	154
Age at recruitment, years	50.4 (9.7)	56.2 (4.4)	52.1 (6.6)	48.3 (8.9)	52.4 (12.6)	49.9 (8.1)	50.1 (11.7)	47.6 (4.3)	47.5 (8.4)	55.3 (8.0)	47.3 (14.4)
Body mass index (kg m^−2^)	24.9 (4.4)	25.6 (4.4)	23.0 (3.4)	25.6 (4.7)	28.5 (5.2)	25.6 (4.3)	25.1 (4.2)	24.4 (3.8)	28.1 (4.7)	25.1 (4.2)	24.2 (4.2)
Current smokers (%)	19.7	31.4	8.8	18.6	17.5	26.3	28.0	31.7	19.4	24.6	11.0
											
*Unilateral ovariectomy (%)*											
Yes	3.4	6.0	3.5	5.2	1.8	4.3	5.6	NR	2.9	NR	3.1
No	77.7	92.9	91.9	94.7	97.8	95.3	92.5	NR	97.0	NR	93.5
Missing	18.9	1.1	4.6	0.1	0.4	0.4	1.9	NR	0.1	NR	3.4
											
*Hysterectomy (%)*											
Yes	7.9	11.4	7.6	12.5	3.1	5.5	15.3	4.7	3.3	NR	10.0
No	82.6	88.5	89.6	87.5	96.9	94.3	84.6	70.9	96.6	NR	88.7
Missing	9.5	0.1	2.8	0	0	0.2	0.1	24.4	0.1	NR	1.3
											
*Oral contraceptive use (%)*											
Ever	58.9	58.2	61.6	81.7	9.8	41.4	73.8	64.1	42.9	51.1	66.5
Never	40.6	40.8	38.2	18.2	90.2	58.4	26.1	35.9	57.0	48.2	32.0
Missing	0.5	1.0	0.2	0.1	0	0.2	0.1	0	0.1	0.7	1.5
											
Duration (years) of OC use^a^	5 (2–10)	6 (2–12)	5 (2–10)	10 (5–30)	1 (1–3)	2 (1–5)	10 (5–15)	3 (1–7)	2 (1–5)	7 (3–14)	6 (3–10)
Age at first OC use (years)^a^	25.4 (6.7)	27.7 (5.7)	28.6 (6.5)	NR	26.5 (6.0)	28.3 (6.8)	25.6 (7.3)	22.4 (4.1)	NR	26.5 (7.3)	22.0 (5.8)
											
*FTP (%)* b											
Ever	79.6	88.3	83.7	85.4	89.5	86.9	48.7	91.5	88.6	78.9	66.9
Never	13.6	11.4	8.8	14.4	10.3	13.1	6.9	8.5	10.5	9.1	31.3
Missing	6.8	0.3	7.5	0.2	0.2	0	44.4	0	0.9	12.0	1.8
Number of FTP^c^	2.3 (1.0)	2.2 (0.9)	2.3 (0.9)	2.0 (0.9)	2.4 (1.1)	2.1 (0.9)	2.7 (1.3)	2.4 (0.9)	2.7 (1.3)	2.2 (1.0)	2.3 (1.0)
Age at first FTP (years)^c^	24.9 (4.4)	23.8 (4.2)	24.9 (4.0)	24.3 (4.4)	24.2 (4.7)	25.8 (4.3)	25.6 (4.0)	24.1 (4.5)	24.7 (3.9)	24.7 (4.6)	26.0 (4.7)
											
*Incomplete pregnancy (%)* d											
Ever	27.7	40.7	35.6	37.0	58.2	36.8	14.6	NR	24.6	15.9	23.4
Never	44.1	50.7	49.9	51.8	35.0	53.0	34.7	NR	65.0	54.2	47.6
Missing	28.2	8.6	14.5	11.2	6.8	10.2	50.7	NR	10.4	29.9	29.0
											
*Breastfeeding (%)* c											
Ever	81.8	91.7	69.5	84.4	87.3	83.5	81.0	92.8	88.7	69.6	80.7
Never	14.7	6.8	27.9	15.3	11.3	16.0	18.4	5.7	10.8	3.6	13.7
Missing	3.5	1.5	2.6	0.3	1.4	0.5	0.6	1.5	0.5	26.8	5.6
Breastfeeding duration^e^	6 (3–13)	7 (4–12)	5 (2–7)	3 (1–8)	11 (4–24)	7 (3–12)	5 (2–9)	12 (6–19)	9 (4–16)	8 (4–13)	7 (2–15)
											
Age at menarche (years)	13.1 (1.5)	13.7 (1.6)	12.9 (1.4)	13.2 (1.5)	13.2 (1.7)	12.6 (1.5)	13.3 (1.6)	13.3 (1.4)	12.9 (1.6)	13.5 (1.5)	12.9 (1.6)
Age at menopause (years)^f^	50 (46–52)	50 (47–52)	50 (48–53)	50 (46–52)	49 (45–52)	50 (47–52)	50 (46–52)	48 (46–50)	50 (46–52)	50 (47–52)	50 (45–52)
											
*Menopausal status (%)*											
Premenopausal	35.2	7.6	26.9	47.9	38.8	40.9	34.3	35.3	56.6	7.2	50.7
Perimenopausal	19.3	19.4	26.1	13.3	7.2	16.1	18.2	35.3	9.8	25.7	10.6
Postmenopausal	45.5	73.0	47.0	38.8	54.0	43.0	47.5	29.4	33.6	67.1	38.7
Total menstrual lifespan^g^	33 (27–37)	32 (25–36)	35 (30–38)	30 (22–35)	33 (29–37)	35 (31–38)	30 (23–35)	32 (28–35)	33 (30–36)	33 (27–36)	33 (27–37)

Abbreviations: EPIC=European Prospective Investigation into Cancer and Nutrition; FTP=full-term pregnancy; NR=not reported; OC=oral contraceptive.

a Among ever OC users only.

b A FTP is defined as a live or stillbirth.

c Among women with a FTP only.

d An incomplete pregnancy is defined as an induced or spontaneous abortion.

e Among women with a FTP who ever breastfed only; in months.

f Among postmenopausal women only.

g Calculated in years as (age at menopause–age at menarche–duration of OC use–cumulative duration of FTP) in postmenopausal women only.

**Table 2:** HR and 95% CI for oral contraceptive use and ovarian cancer risk in the EPIC cohort

**Variable**	**Cases/non-cases**	**Age- and centre- stratified HR (95% CI)**	**Multivariable-adjusted HR (95% CI)** a
*OC use*			
Never	453/132 622	1.00 (ref.)	1.00 (ref.)
Ever	418/192 401	0.84 (0.72–0.98)	0.86 (0.73–1.00)
			
*Duration of OC use, years* b			
⩽1	96/35 185	1.00 (ref.)	1.00 (ref.)
2–4	109/41 341	1.04 (0.78–1.37)	1.05 (0.79–1.38)
5–9	83/41 723	0.79 (0.59–1.07)	0.80 (0.59–1.08)
⩾10	90/57 246	0.56 (0.42–0.76)	0.55 (0.41–0.75)
*P*-trend		<0.01	<0.01
Per 5 years		0.88 (0.82–0.94)	0.87 (0.82–0.93)
			
*Age at first OC use* b			
<25	115/75 496	1.00 (ref.)	1.00 (ref.)
25–29	81/34 282	1.03 (0.75–1.42)	1.04 (0.76–1.44)
30–34	73/20 495	1.34 (0.92–1.94)	1.36 (0.94–1.97)
⩾35	55/15 838	1.21 (0.78–1.87)	1.20 (0.78–1.86)
*P*-trend		0.23	0.23

Abbreviations: CI=confidence interval; EPIC=European Prospective Investigation into Cancer and Nutrition; HR=hazard ratio; OC=oral contraceptive; ref.=reference.

a From a Cox proportional hazards model stratified by EPIC-participating centre and age at recruitment, and adjusted for smoking status (never, former, current), body mass index (kg m^−2^, continuous), unilateral ovariectomy (no, yes), simple hysterectomy (no, yes), menopausal hormone therapy (never, former, current use: oestrogen-only, oestrogen plus progestin, other formulation), age at menarche (<12, 12, 13, 14, ⩾15 years), number of full-term pregnancies (0, 1, 2, 3, ⩾4) and age at menopause (premenopausal, perimenopausal, postmenopausal: ⩽45, 46–50, 51–52, >52 years).

b Among ever OC users only.

**Table 3:** HR and 95% CI for duration of oral contraceptive use and ovarian cancer risk by BMI and menopausal status in the EPIC cohort

	**BMI <24 kg m^−2^**		**BMI ⩾24 kg m^−2^**		**Pre/perimenopausal**		**Postmenopausal**	
**Variable**	**Cases/non-cases**	**HR (95% CI)** a	**Cases/non-cases**	**HR (95% CI)** a	**Cases/non-cases**	**HR (95% CI)** b	**Cases/non-cases**	**HR (95% CI)** b
*Duration of OC use, years* c								
⩽1	52/17 673	1.00 (ref.)	44/17 512	1.00 (ref.)	54/22 645	1.00 (ref.)	42/12 540	1.00 (ref.)
2–4	58/22 652	0.93 (0.63–1.36)	51/18 689	1.19 (0.79–1.79)	58/28 756	0.88 (0.60–1.28)	51/12 585	1.27 (0.84–1.92)
5–9	38/23 922	0.57 (0.37–0.87)	45/17 801	1.13 (0.73–1.74)	38/29 806	0.55 (0.36–0.85)	45/11 917	1.18 (0.77–1.81)
⩾10	39/30 969	0.38 (0.25–0.59)	51/26 277	0.80 (0.52–1.23)	41/37 013	0.41 (0.26–0.63)	49/20 233	0.76 (0.49–1.17)
*P*-trend		<0.01		0.24		<0.01		0.14
*P*-interaction				0.01				0.02
Per 5 years		0.80 (0.71–0.89)		0.94 (0.86–1.02)		0.83 (0.75–0.92)		0.91 (0.84–1.00)
*P*-interaction (continuous)				0.02				0.13

Abbreviations: BMI=body mass index; CI=confidence interval; EPIC=European Prospective Investigation into Cancer and Nutrition; HR=hazard ratio; OC=oral contraceptive; ref.=reference.

a From a Cox proportional hazards model stratified by EPIC-participating centre and age at recruitment, and adjusted for smoking status (never, former, current), unilateral ovariectomy (no, yes), simple hysterectomy (no, yes), menopausal hormone therapy (never, former, current use: oestrogen-only, oestrogen plus progestin, other formulation), age at menarche (<12, 12, 13, 14, ⩾15 years), number of full-term pregnancies (0, 1, 2, 3, ⩾4) and age at menopause (premenopausal, perimenopausal, postmenopausal: ⩽45, 46–50, 51–52, >52 years).

b From a Cox proportional hazards model stratified by EPIC-participating centre and age at recruitment, and adjusted for smoking status (never, former, current), body mass index (kg m^−2^, continuous), unilateral ovariectomy (no, yes), simple hysterectomy (no, yes), age at menarche (<12, 12, 13, 14, ⩾15 years) and number of full-term pregnancies (0, 1, 2, 3, ⩾4).

c Among ever OC users only.

**Table 4:** HR and 95% CI for parity, other reproductive variables and ovarian cancer risk in the EPIC cohort

**Variable**	**Cases/non-cases**	**Age- and centre- stratified HR (95% CI)**	**Multivariable-adjusted HR (95% CI)** a
*FTP* b			
Never	133/44 328	1.00 (ref.)	1.00 (ref.)
Ever	689/259 740	0.70 (0.58–0.85)	0.71 (0.59–0.87)
			
*Number of FTP*			
Never	133/44 328	1.00 (ref.)	1.00 (ref.)
1	135/47 766	0.79 (0.62–1.01)	0.80 (0.63–1.02)
2	335/125 984	0.73 (0.59–0.89)	0.74 (0.61–0.91)
3	149/59 703	0.63 (0.49–0.80)	0.64 (0.50–0.81)
⩾4	70/26 287	0.61 (0.45–0.82)	0.62 (0.46–0.83)
*P*-trend		<0.01	<0.01
			
*Number of FTP* c			
1	135/47 766	1.00 (ref.)	1.00 (ref.)
2	335/125 984	0.93 (0.76–1.14)	0.94 (0.77–1.15)
3	149/59 703	0.80 (0.63–1.02)	0.81 (0.64–1.02)
⩾4	70/26 287	0.77 (0.57–1.04)	0.77 (0.57–1.04)
*P*-trend		0.03	0.03
Per FTP		0.92 (0.85–0.99)	0.92 (0.85–0.99)
			
*Age at first FTP (years)* c			
⩽20	97/38 591	1.00 (ref.)	1.00 (ref.)
21–23	170/69 656	0.91 (0.71–1.17)	0.92 (0.72–1.19)
24–25	145/50 567	1.05 (0.81–1.36)	1.07 (0.82–1.39)
26–30	208/73 523	1.03 (0.80–1.31)	1.05 (0.81–1.34)
>30	64/26 419	0.91 (0.66–1.26)	0.93 (0.67–1.29)
*P*-trend		0.79	0.69
			
*Incomplete pregnancy* d			
Never	413/143 841	1.00 (ref.)	1.00 (ref.)
Ever	237/90 352	1.00 (0.85–1.18)	1.00 (0.85–1.18)
			
*Breastfeeding* c			
Never	115/38 087	1.00 (ref.)	1.00 (ref.)
Ever	543/212 490	0.86 (0.69–1.06)	0.86 (0.70–1.07)
			
*Duration of breastfeeding, months* c			
⩽1 (Including never)	176/58 968	1.00 (ref.)	1.00 (ref.)
2–6	205/85 202	0.83 (0.68–1.02)	0.84 (0.68–1.03)
7–12	134/50 185	0.90 (0.71–1.13)	0.91 (0.72–1.14)
>13	139/54 475	0.87 (0.68–1.11)	0.88 (0.69–1.13)
*P*-trend		0.38	0.45
			
*Age at menarche (years)*			
<12	145/48 285	1.00 (ref.)	1.00 (ref.)
12	159/69 012	0.75 (0.60–0.94)	0.76 (0.61–0.96)
13	208/83 793	0.77 (0.62–0.96)	0.79 (0.64–0.98)
14	192/70 040	0.79 (0.64–0.99)	0.82 (0.66–1.02)
⩾15	161/51 355	0.82 (0.65–1.04)	0.85 (0.68–1.08)
*P*-trend		0.29	0.46
Per year		1.00 (0.95–1.04)	1.00 (0.96–1.05)
			
*Age at menopause (years)* e			
⩽45	75/23 730	1.00 (ref.)	1.00 (ref.)
46–50	172/46 885	1.14 (0.87–1.50)	1.12 (0.84–1.49)
51–52	75/19 790	1.11 (0.80–1.54)	1.08 (0.77–1.52)
>52	120/22 831	1.51 (1.12–2.03)	1.46 (1.06–1.99)
*P*-trend		<0.01	0.02
Per year		1.02 (1.00–1.05)	1.02 (1.00–1.05)
			
*Menopausal status*			
Pre/perimenopausal	331/178 107	1.00 (ref.)	1.00 (ref.)
Postmenopausal	547/148 411	0.92 (0.73–1.16)	0.87 (0.69–1.10)
			
*Total menstrual lifespan, years* f			
⩽27	75/25 330	1.00 (ref.)	1.00 (ref.)
28–32	71/22 627	1.05 (0.76–1.46)	1.07 (0.77–1.49)
33–36	121/28 006	1.42 (1.06–1.91)	1.44 (1.07–1.95)
>36	135/27 225	1.56 (1.16–2.10)	1.57 (1.16–2.13)
*P*-trend		<0.01	<0.01
Per year		1.02 (1.01–1.04)	1.02 (1.01–1.04)

Abbreviations: CI=confidence interval; EPIC=European Prospective Investigation into Cancer and Nutrition; FTP=full-term pregnancy; HR=hazard ratio; ref.=reference.

a From a Cox proportional hazards model stratified by EPIC-participating centre and age at recruitment, and adjusted for smoking status (never, former, current), body mass index (kg m^**−**2^, continuous), unilateral ovariectomy (no, yes), simple hysterectomy (no, yes), menopausal hormone therapy (never, former, current use: oestrogen-only, oestrogen plus progestin, other formulation), duration of oral contraceptive use (never, ever: ⩽1, 2–4, 5–9, ⩾10 years), age at menarche (<12, 12, 13, 14, ⩾15 years) and age at menopause (premenopausal, perimenopausal, postmenopausal: ⩽45, 46–50, 51–52, >52 years). The models for incomplete pregnancy, age at menarche and menopause were further adjusted for number of FTPs (0, 1, 2, 3, ⩾4). The model for menopausal status was not adjusted for menopausal hormone therapy and age at menopause. The model for total menstrual lifespan was not adjusted for age at menopause, age at menarche, duration of oral contraceptive use and number of FTPs.

b A FTP is defined as a live or stillbirth.

c Among women with a FTP.

d An incomplete pregnancy is defined as an induced or spontaneous abortion.

e Among postmenopausal women only.

f Calculated as (age at menopause–age at menarche–duration of oral contraceptive use–cumulative duration of FTP) in postmenopausal women only.

## References

[bib1] Adami HO, Hsieh CC, Lambe M, Trichopoulos D, Leon D, Persson I, Ekbom A, Janson PO (1994) Parity, age at first childbirth, and risk of ovarian cancer. Lancet 344: 1250–1254 7967985 10.1016/s0140-6736(94)90749-8

[bib2] Albrektsen G, Heuch I, Kvale G (1996) Reproductive factors and incidence of epithelial ovarian cancer: a Norwegian prospective study. Cancer Causes Control 7: 421–427 8813430 10.1007/BF00052668

[bib3] American Cancer Society (2007) Global Cancer Facts and Figures 2007. American Cancer Society: Atlanta

[bib4] Beral V, Doll R, Hermon C, Peto R, Reeves G (2008) Ovarian cancer and oral contraceptives: collaborative reanalysis of data from 45 epidemiological studies including 23 257 women with ovarian cancer and 87 303 controls. Lancet 371: 303–314 18294997 10.1016/S0140-6736(08)60167-1

[bib5] Booth M, Beral V, Smith P (1989) Risk factors for ovarian cancer: a case-control study. Br J Cancer 60: 592–598 2679848 10.1038/bjc.1989.320 PMC2247100

[bib6] Braem MG, Onland-Moret NC, van den Brandt PA, Goldbohm RA, Peeters PH, Kruitwagen RF, Schouten LJ (2010) Reproductive and hormonal factors in association with ovarian cancer in the Netherlands Cohort Study. Am J Epidemiol 172: 1181–1189 20861144 10.1093/aje/kwq264 PMC2970782

[bib7] Cramer DW, Welch WR (1983) Determinants of ovarian cancer risk. II. Inferences regarding pathogenesis. J Natl Cancer Inst 71: 717–721 6578367

[bib8] Cramer DW, Xu H (1995) Epidemiologic evidence for uterine growth factors in the pathogenesis of ovarian cancer. Ann Epidemiol 5: 310–314 8520714 10.1016/1047-2797(94)00098-e

[bib9] Danforth KN, Tworoger SS, Hecht JL, Rosner BA, Colditz GA, Hankinson SE (2007) Breastfeeding and risk of ovarian cancer in two prospective cohorts. Cancer Causes Control 18: 517–523 17450440 10.1007/s10552-007-0130-2

[bib10] Dossus L, Allen N, Kaaks R, Bakken K, Lund E, Tjonneland A, Olsen A, Overvad K, Clavel-Chapelon F, Fournier A, Chabbert-Buffet N, Boeing H, Schütze M, Trichopoulou A, Trichopoulos D, Lagiou P, Palli D, Krogh V, Tumino R, Vineis P, Mattiello A, Bueno-de-Mesquita HB, Onland-Moret NC, Peeters PH, Dumeaux V, Redondo ML, Duell E, Sanchez-Cantalejo E, Arriola L, Chirlaque MD, Ardanaz E, Manjer J, Borgquist S, Lukanova A, Lundin E, Khaw KT, Wareham N, Key T, Chajes V, Rinaldi S, Slimani N, Mouw T, Gallo V, Riboli E (2010) Reproductive risk factors and endometrial cancer: the European Prospective Investigation into Cancer and Nutrition. Int J Cancer 127: 442–451 19924816 10.1002/ijc.25050

[bib11] Fathalla MF (1971) Incessant ovulation – a factor in ovarian neoplasia? Lancet 2: 163 4104488 10.1016/s0140-6736(71)92335-x

[bib12] Gwinn ML, Lee NC, Rhodes PH, Layde PM, Rubin GL (1990) Pregnancy, breast feeding, and oral contraceptives and the risk of epithelial ovarian cancer. J Clin Epidemiol 43: 559–568 2348208 10.1016/0895-4356(90)90160-q

[bib13] Hankinson SE, Colditz GA, Hunter DJ, Willett WC, Stampfer MJ, Rosner B, Hennekens CH, Speizer FE (1995) A prospective study of reproductive factors and risk of epithelial ovarian cancer. Cancer 76: 284–290 8625104 10.1002/1097-0142(19950715)76:2<284::aid-cncr2820760219>3.0.co;2-5

[bib14] Harlow BL, Cramer DW, Bell DA, Welch WR (1992) Perineal exposure to talc and ovarian cancer risk. Obstet Gynecol 80: 19–26 1603491

[bib15] Jordan SJ, Siskind V, Green AC, Whiteman DC, Webb PM (2010) Breastfeeding and risk of epithelial ovarian cancer. Cancer Causes Control 21: 109–116 19779839 10.1007/s10552-009-9440-x

[bib16] Jordan SJ, Webb PM, Green AC (2005) Height, age at menarche, and risk of epithelial ovarian cancer. Cancer Epidemiol Biomarkers Prev 14: 2045–2048 16103459 10.1158/1055-9965.EPI-05-0085

[bib17] Kvale G, Heuch I, Nilssen S, Beral V (1988) Reproductive factors and risk of ovarian cancer: a prospective study. Int J Cancer 42: 246–251 3403067 10.1002/ijc.2910420217

[bib18] Lau KM, Mok SC, Ho SM (1999) Expression of human estrogen receptor-alpha and -beta, progesterone receptor, and androgen receptor mRNA in normal and malignant ovarian epithelial cells. Proc Natl Acad Sci USA 96: 5722–5727 10318951 10.1073/pnas.96.10.5722 PMC21927

[bib19] Mogren I, Stenlund H, Hogberg U (2001) Long-term impact of reproductive factors on the risk of cervical, endometrial, ovarian and breast cancer. Acta Oncol 40: 849–854 11859985 10.1080/02841860152703481

[bib20] Moorman PG, Palmieri RT, Akushevich L, Berchuck A, Schildkraut JM (2009) Ovarian cancer risk factors in African-American and white women. Am J Epidemiol 170: 598–606 19605513 10.1093/aje/kwp176 PMC2732987

[bib21] Mukherjee K, Syed V, Ho SM (2005) Estrogen-induced loss of progesterone receptor expression in normal and malignant ovarian surface epithelial cells. Oncogene 24: 4388–4400 15806153 10.1038/sj.onc.1208623

[bib22] Negri E, Franceschi S, Tzonou A, Booth M, La Vecchia C, Parazzini F, Beral V, Boyle P, Trichopoulos D (1991) Pooled analysis of 3 European case-control studies: I. Reproductive factors and risk of epithelial ovarian cancer. Int J Cancer 49: 50–56 1874569 10.1002/ijc.2910490110

[bib23] Pike MC, Pearce CL, Peters R, Cozen W, Wan P, Wu AH (2004) Hormonal factors and the risk of invasive ovarian cancer: a population-based case-control study. Fertil Steril 82: 186–195 15237010 10.1016/j.fertnstert.2004.03.013

[bib24] Piper JM, Kennedy DL (1987) Oral contraceptives in the United States: trends in content and potency. Int J Epidemiol 16: 215–221 3301706 10.1093/ije/16.2.215

[bib25] Purdie D, Green A, Bain C, Siskind V, Ward B, Hacker N, Quinn M, Wright G, Russell P, Susil B (1995) Reproductive and other factors and risk of epithelial ovarian cancer: an Australian case-control study. Survey of Women's Health Study Group. Int J Cancer 62: 678–684 7558414 10.1002/ijc.2910620606

[bib26] Riboli E, Hunt KJ, Slimani N, Ferrari P, Norat T, Fahey M, Charrondiere UR, Hemon B, Casagrande C, Vignat J, Overvad K, Tjønneland A, Clavel-Chapelon F, Thiébaut A, Wahrendorf J, Boeing H, Trichopoulos D, Trichopoulou A, Vineis P, Palli D, Bueno-De-Mesquita HB, Peeters PH, Lund E, Engeset D, González CA, Barricarte A, Berglund G, Hallmans G, Day NE, Key TJ, Kaaks R, Saracci R (2002) European Prospective Investigation into Cancer and Nutrition (EPIC): study populations and data collection. Public Health Nutr 5: 1113–1124 12639222 10.1079/PHN2002394

[bib27] Riman T, Dickman PW, Nilsson S, Correia N, Nordlinder H, Magnusson CM, Persson IR (2002) Risk factors for invasive epithelial ovarian cancer: results from a Swedish case-control study. Am J Epidemiol 156: 363–373 12181107 10.1093/aje/kwf048

[bib28] Riman T, Nilsson S, Persson IR (2004) Review of epidemiological evidence for reproductive and hormonal factors in relation to the risk of epithelial ovarian malignancies. Acta Obstet Gynecol Scand 83: 783–795 15315588 10.1111/j.0001-6349.2004.00550.x

[bib29] Risch HA (1998) Hormonal etiology of epithelial ovarian cancer, with a hypothesis concerning the role of androgens and progesterone. J Natl Cancer Inst 90: 1774–1786 9839517 10.1093/jnci/90.23.1774

[bib30] Risch HA, Marrett LD, Howe GR (1994) Parity, contraception, infertility, and the risk of epithelial ovarian cancer. Am J Epidemiol 140: 585–597 7942759 10.1093/oxfordjournals.aje.a117296

[bib31] Schildkraut JM, Cooper GS, Halabi S, Calingaert B, Hartge P, Whittemore AS (2001) Age at natural menopause and the risk of epithelial ovarian cancer. Obstet Gynecol 98: 85–90 11430962 10.1016/s0029-7844(01)01388-6

[bib32] Schoenfeld D (1982) Partial residuals for the proportional hazards regression model. Biometrika 69: 239–241

[bib33] Stadel BV (1975) Letter: The etiology and prevention of ovarian cancer. Am J Obstet Gynecol 123: 772–774 1200073 10.1016/0002-9378(75)90509-8

[bib34] Syed V, Ulinski G, Mok SC, Yiu GK, Ho SM (2001) Expression of gonadotropin receptor and growth responses to key reproductive hormones in normal and malignant human ovarian surface epithelial cells. Cancer Res 61: 6768–6776 11559549

[bib35] Thorogood M, Villard-Mackintosh L (1993) Combined oral contraceptives: risks and benefits. Br Med Bull 49: 124–139 8324603 10.1093/oxfordjournals.bmb.a072592

[bib36] Titus-Ernstoff L, Perez K, Cramer DW, Harlow BL, Baron JA, Greenberg ER (2001) Menstrual and reproductive factors in relation to ovarian cancer risk. Br J Cancer 84: 714–721 11237375 10.1054/bjoc.2000.1596 PMC2363792

[bib37] Tung KH, Goodman MT, Wu AH, McDuffie K, Wilkens LR, Kolonel LN, Nomura AM, Terada KY, Carney ME, Sobin LH (2003) Reproductive factors and epithelial ovarian cancer risk by histologic type: a multiethnic case-control study. Am J Epidemiol 158: 629–638 14507598 10.1093/aje/kwg177

[bib38] Vachon CM, Mink PJ, Janney CA, Sellers TA, Cerhan JR, Hartmann L, Folsom AR (2002) Association of parity and ovarian cancer risk by family history of breast or ovarian cancer in a population-based study of postmenopausal women. Epidemiology 13: 66–71 11805588 10.1097/00001648-200201000-00011

[bib39] Whiteman DC, Siskind V, Purdie DM, Green AC (2003) Timing of pregnancy and the risk of epithelial ovarian cancer. Cancer Epidemiol Biomarkers Prev 12: 42–46 12540502

[bib40] Whittemore AS, Harris R, Itnyre J (1992) Characteristics relating to ovarian cancer risk: collaborative analysis of 12 US case-control studies. II. Invasive epithelial ovarian cancers in white women. Collaborative Ovarian Cancer Group. Am J Epidemiol 136: 1184–1203 1476141 10.1093/oxfordjournals.aje.a116427

[bib41] Zhang M, Lee AH, Binns CW (2004) Reproductive and dietary risk factors for epithelial ovarian cancer in China. Gynecol Oncol 92: 320–326 14751177 10.1016/j.ygyno.2003.10.025

